# SMARTPHONE OVERUSE AMONG OLDER ADULTS: A SCOPING REVIEW

**DOI:** 10.1093/geroni/igae098.4077

**Published:** 2024-12-31

**Authors:** Jeongeun Choi, Hyangkyu Lee, Seolah Yoon, Hyeonmi Cho

**Affiliations:** Yonsei University, Seoul, Republic of Korea; Yonsei University, Seoul, Republic of Korea; Yonsei University, Seoul, Republic of Korea; Yonsei University, Seoul, Republic of Korea

## Abstract

The rapid advancement of technology and widespread adoption of smartphones have led to increased smartphone usage among older adults. While smartphones offer many benefits, excessive use can lead to health issues, especially for older adults who may experience reduced social interactions and higher levels of anxiety or depressive symptoms. However, most existing research has focused primarily on adolescents and younger adults. This scoping review aimed to investigate the terms and definitions, factors associated with smartphone overuse, and its outcomes in older adults. We searched seven databases, including PubMed, PsycINFO, Embase, Cochrane Library, Web of Science, Scopus, and CINAHL, covering studies published from 2007 to 2024. We followed the Joanna Briggs Institute scoping review methodology, and sixteen studies were selected. Smartphone overuse was characterized by various terms and definitions, including ‘smartphone overdependence’, ‘smartphone addiction’, and ‘problematic smartphone usage’. According to the biopsychosocial model, factors related to smartphone overuse were classified into biological, behavioral, psychological, and social categories. Most commonly reported factors included biological aspects such as age, sex, and BMI; behavioral factors like types of smartphone activities; psychological factors including loneliness, anxiety, and depressive symptoms; and social factors such as income and educational level. Smartphone overuse can negatively affect physical, psychological, and social health, resulting in pain in the musculoskeletal system, sleep disturbances, anxiety, depressive symptoms, and loneliness. Future research should focus on developing standardized definitions and exploring effective interventions to mitigate the negative effects of smartphone overuse in older adults.

